# Sensory Abnormalities in Autism Spectrum Disorders: A Focus on the Tactile Domain, From Genetic Mouse Models to the Clinic

**DOI:** 10.3389/fpsyt.2019.01016

**Published:** 2020-01-28

**Authors:** Luigi Balasco, Giovanni Provenzano, Yuri Bozzi

**Affiliations:** ^1^ Center for Mind/Brain Sciences (CIMeC), University of Trento, Rovereto, Italy; ^2^ Department of Cellular, Computational and Integrative Biology (CIBIO), University of Trento, Trento, Italy; ^3^ CNR Neuroscience Institute, Pisa, Italy

**Keywords:** autism, somatosensory, touch, mouse, behavior

## Abstract

Sensory abnormalities are commonly recognized as diagnostic criteria in autism spectrum disorder (ASD), as reported in the last edition of the Diagnostic and Statistical Manual of Mental Disorder (DSM-V). About 90% of ASD individuals have atypical sensory experiences, described as both hyper- and hypo-reactivity, with abnormal responses to tactile stimulation representing a very frequent finding. In this review, we will address the neurobiological bases of sensory processing in ASD, with a specific focus of tactile sensitivity. In the first part, we will review the most relevant sensory abnormalities detected in ASD, and then focus on tactile processing deficits through the discussion of recent clinical and experimental studies. In the search for the neurobiological bases of ASD, several mouse models have been generated with knockout and humanized knockin mutations in many ASD-associated genes. Here, we will therefore give a brief overview of the anatomical structure of the mouse somatosensory system, and describe the somatosensory abnormalities so far reported in different mouse models of ASD. Understanding the neurobiological bases of sensory processing in ASD mouse models may represent an opportunity for a better comprehension of the mechanisms underlying sensory abnormalities, and for the development of novel effective therapeutic strategies.

## Introduction: Altered Sensory Reactivity in ASD

Individuals diagnosed with autism spectrum disorder (ASD) show deficits in social interaction and communication (developing, understanding and maintaining relationships) and repetitive/stereotyped behaviors, with different degree of severity, and sensory issues (1). However, it is interesting how typical cognitive difficulties of ASD are often associated with alterations in the perception of the external world. It is estimated that about 90% of individuals diagnosed with ASD have atypical sensory experiences (2). Differences in the sensory profile of ASD subjects are confirmed across lifespan (3, 4) and cross-culturally (5). Indeed, this trait is nowadays recognized in the DSM-V as hyper/hypo reactivity to sensory stimuli, demonstrating its primary importance in the description of the syndrome.

The correlation among autism and sensory deficits is not new. Formerly, (6) one of the first to describe autism, included different atypical sensory behaviors in his analysis (including heightened sensitivity to noise and touch, attraction to visual patterns and spinning objects, finger-stimming in front of the eyes) although considering them as a secondary phenomenon that occurs in parallel to the primary phenomenon (6).

(7) were the first instead to describe, in a case report study, a group of children who were particularly reactive to “unusual sensitivities” (low intensities of stimulation) in several sensory modalities (7). They hypothesized that an early developmental onset of sensitivity to sensory stimuli would cause social withdrawal in childhood.

Based on the Freud’s “protective barrier against stimuli” (Reizschutz), they proposed that these children eventually succeed in building defending strategies to protect themselves from sensory overload, which would result in developmental distortions typical of autistic conditions. Eveloff described different behavioral difficulties faced by autistic children (8). He hypothesized that altered sensory processing is the effect of the lack of early experiences of environmental stimuli therefore interfering with the development of self-representations in autism. Psychologist Lorna Wing noted the “detail-oriented” behavior of autistic children, showing that they have significantly more sensory processing abnormalities than both typically developing (TD) children and children with Down’s syndrome (9). She was the first to suggest to include abnormal sensory perceptual features as a proper diagnostic tool into ‘basic impairments in autism’. However, this was not included in the first diagnostic criteria for autism by DSM in 1980 (10). Ornitz extended the concept of autism as a sensory and information processing disorder (11). He suggested that autism could be highlighted in young children looking at abnormal behaviors caused by perceptual differences (12). Nonetheless, there have been detractors of this theory and the strength of sensory features in autism has been put under scrutiny during the past decades. As an example, Richer strongly argued against the sensory theory of autism stating it was “incoherent and instable” (13). He, instead, stated that the autistic children’s incompetence in language and symbol use was mainly due to their avoidance behavior in communication interactions. Similarly, Rutter proposed language deficits as the base of autistic syndrome, distancing himself from the sensory theory (14). Another line of research came in parallel from the field of occupational therapy (OT). (15) formulated the theory of sensory integration (SI) dysfunction to describe several neurological disorders including autism. This theory tried to relate sensory processing deficits with behavioral abnormalities and had the merit to define SI in terms of behavioral responses identifying for example tactile defensiveness and fight-or-flight reactions. More recently, OT researchers have suggested to consider the use of the term “sensory processing disorder” as comprising three primary groups (sensory modulation disorder, sensory discrimination disorder and sensory-based motor disorder) and the subtypes found within each group (16). However, sensory processing disorder is not still considered as a disorder *per se*. With Rutter, the theory of “social-perception” took hold in 1983 (17). He concluded the sensory symptoms found in the autistic population were the result of deficits in social cognition. It is not the processing of a sensory stimulus per se that creates difficulties in the autistic subject, but rather the processing of stimuli of emotional nature (i.e., those that possess a social content). Finally, in 2013 sensory processing deficits were included for the first time among the international diagnostic criteria of autism in the revision of Diagnostic and Statistical Manual of Mental Disorders (DSM-V) (1). From a clinical point of view, sensory deficits are documented already in the 6th month of life of infants later diagnosed with autism (18, 19). This gives us a dual information, firstly that sensory symptoms anticipate social and communication deficits (19), and secondly that abnormal sensory traits could be predictive of the autistic condition (20). This appears strikingly evident when considering that not only the vast majority of individual diagnosed with autism experience atypical reactivity to sensory stimuli (2, 21), but also that this affect every sensory modality: smell (22, 23), taste (24), audition (25), vision (26), and touch (27, 28). It seems clear that understanding the neurobiological bases underlying these sensory processing deficits represents a new challenge for ASD research, specifically aiming to identify early biomarkers and novel possible therapeutic strategies for these disorders.

Here, we will describe sensory defects in ASD, specifically focusing on altered tactile sensitivity. An in-depth analysis of somatosensory defects detected in mouse lines harboring mutations in ASD-relevant genes will also be presented. The aim of this review is to highlight the contribution of animal model studies in our understanding of the neurobiological bases of altered sensory sensitivity in ASD.

## Smell, Taste, Auditory, and Visual Deficits in ASD

### Smell

The sense of smell in ASD is poorly investigated. Nonetheless, a parent-report study pointed out how the most pronounced sensory symptom to dissociate ASD children from children with other developmental disorders are de facto taste and smell abnormal responses (29). Furthermore, it has been reported that almost 40% of ASD children with sensory abnormalities exhibit an altered smell and taste perception (30). Children and adolescents aged between 10 and 18 years showed impaired olfactory identification, but typical odor detection (31). Another study with ASD children aged from 5 to 9 years showed no differences in olfactory identification compared with controls, however older children were less accurate than younger ones at identifying odors (32). In the follow-up study (5 years later), the same ASD individuals had developed odor identification impairments (33). A more recent study confirmed that ASD children present impaired odor identification but normal odor detection compared to control participants (22). However, a clear picture of how and when altered olfaction occurs in the ASD cascade has not yet emerged (22). Considering the possible influence of language in the common odor task, Rosenkrantz and coworkers suggested to use olfactory sniffing as a language and task-free measure of autism and its severity. Since sniffs are automatically modulated (vigorous sniffs for pleasant and truncated sniffs for unpleasant odors), these authors found that children with autism had a profound altered sniff response, sniffing equally regardless of odor valence (for example taking vigorous sniffs of rotten fish odor) compared to typically developing controls (23). These authors also found that this difference persisted despite equal reported odor perception and allowed for 81% correct ASD classification based on the sniff response alone. Moreover, they found that increasingly aberrant sniffing was associated with increasingly severe ASD, proposing it as a novel ASD biomarker (23).

### Taste

There are few studies that deal with taste processing in ASD. However, it has been reported that children with ASD eat a smaller variety of food (e.g., less vegetables, fruits, dairy) regardless of texture, and refuse more food than typical developing children (34, 35). Bennetto and colleagues found a lower accuracy in taste discrimination for sour and bitter tastes, but similar identification for sweet and salty tastes, in adolescents (10–18 years) with ASD compared with control peers (31). Similar results were found in adults, except for sweet taste which was also impaired in those adults with ASD (24). A possible explanation for these taste identification differences in ASD might stem from the restricted diets of ASD patients that could alternatively explain why adolescents and adults are less accurate in identifying tastes (24). Abnormalities at the level of peripheral receptors and their transduction cascades could lead to taste impairments (36). Another view focuses on central rather than peripheral mechanisms (31). Indeed, central areas such as the thalamus, insula and cingulate cortex are involved in taste discrimination (37), and areas including the thalamus have been shown to be reduced in size in individuals with ASD (38). Thus, the difference in taste processing might be the result of atypical activity in these areas. However, further investigation is needed to understand whether ASD is associated with taste sensory defects at a peripheral or central level.

### Audition

Sensory processing abnormalities have also been observed in the auditory domain. Indeed, children with autism often show difficulty in discerning two occurring tones when presented closely (39). In addition, a delayed evoked neural response compared to TD children have also been documented (40, 41). This latency has been observed in response to pure tones as well as to complex social stimuli (for example sound produced by speech) (42) and has been proposed as predictive of autism symptom severity (43). Although evidences for sensory processing deficits are more and more abundant in ASD literature, there are several reports that highlight enhanced perceptual strengths in response to specific sensory stimuli. As an example, individuals with ASD show superior abilities in pitch discrimination and in categorization compared to controls (25). In an effort to bring together all these findings, it has been suggested that perceptual capabilities may be subject to the nature and complexity of the sensory stimuli, with impairments associated with more complex stimuli and enhancements seen more often with simple stimuli (44, 45).

### Vision

Over the years, there have been many studies investigating different aspects of visual perception in ASD. Defective retinal function has been described in ASD patients (46–49). Enhanced visual evoked potentials (VEP) in response to high spatial frequencies have been found in visual brain areas of ASD children, while unaffected control children generally responded to visual stimuli with low spatial frequency (50). Other studies showed that visual perception in ASD is more detail-oriented, suggesting that primary visual processing might contribute to social and communication deficits in ASD (51–54). It is generally accepted that individuals with ASD “see” and process the world differently, having a strong detail oriented ability in expense to global processing (55). ASD individuals are faster in detecting single details in Embedded Figure Tasks (EFT- a task in which participants are asked to find a target figure hidden in a larger image), being less susceptible to distractors (56–60). Moreover, gaze patterns from individuals with autism show a preference for scene regions of high pixel-level saliency compared with object-level saliency or semantic-level saliency scenes in passive viewing of naturalistic scenes (61). This means that they favor regions of the scene that are related with contrast, color, or orientation (pixel-level) rather than related with size, density, or contour complexity of objects (object-level) or related with text, tools, or faces (semantic-level). How individuals with ASD have this detail-oriented visual ability is still under debate. Moreover, further complexity is given from the fact that in autism, basic measures of visual sensitivity such as visual acuity (59, 62), contrast discrimination (63, 64), and orientation processing (65, 66) are all comparable with normal developing children. Conversely to the static stimuli, ASD individuals show atypical processing of dynamic visual stimuli (67, 68). Indeed, ASD subjects show an impaired global motion perception in discerning the direction of a cloud of moving dots (69, 70), even though the detection thresholds for local motion appear to be typical (71), or ever superior in ASD (72, 73).

## Tactile Senitivity in ASD

The typical description of sensory processing abnormalities falls in the terminology of “over-responsiveness”, “under-responsiveness”, and “failure to habituate”. Over-responsiveness, also called hyper-sensitivity, refers to children being more “reactive” to sensory stimulation compared to controls (74, 75), often associated with negative emotion or active avoidance of stimulation. However, the terminology used in clinical reports and questionnaires often fails in separating “over-responsiveness” from “impaired habituation”. Moreover, it is unclear whether this refers to hyper-excitability of sensory cortex or the expression of negative emotions to tactile stimulation. Conversely, under-responsiveness, also called hypo-sensitivity, is characterized by reduced reactivity to sensory stimulation and sensory seeking (75). Both over- and under-responsiveness then fall under the general term of tactile defensiveness (76), which describes both abnormal emotional responses to tactile stimulation as well as withdrawal/avoidance of a stimulation.

The vast majority of studies investigating tactile dysfunction have traditionally focused on parent and teacher reports and questionnaires. These studies, although informative, lack objectivity in the strict sense since they are based on subjective assessments of both behavioral and emotional responses to touch (77). Only recent works addressed the study of tactile abnormalities through a psychophysics approach, aiming to reduce the degree of subjectivity and to highlight neurophysiological underpinnings of this phenomenon.

As reported in a recent review (77), a number of studies have described tactile abnormalities using sensory profiles and parents reports (78–81). By using two parent-report measures, the Short Sensory Profile (SSP) and the Autism Diagnostic Interview-Revised (ADI-R), and a clinical observation with the Autism Diagnostic Observation Scale (ADOS), Rogers et al. compared sensory profiles in toddlers with ASD and typically developing controls and with other groups of developmental delay such as Fragile-X children and children with Down syndrome. They found significantly elevated levels of sensory symptoms in children with autism compared with both children with typical development and those with delayed development of the same mental age. In particular, children with autism obtained significantly higher scores of tactile sensitivity and auditory filtering than children in the developmental delay and controls. Moreover, they observed a correlation between abnormal sensitivity and adaptive behaviors. They also found no meaningful relationships between social-communicative scores and sensory scores in children with mixed developmental delays, or the typically developing children. The explanation of the authors is that since sensory symptoms are not in general a peculiarity of autism, they could represent an additional primary impairment rather than an autism-specific impairment. Moreover, they found that sensory scores (including tactile scores) did not correlate with either developmental levels or with ratio IQ scores for any group except the children with Fragile-X syndrome. Increased sensory scores were associated with clinical diagnosis rather than with IQ or immature developmental levels (78). Other tests including the Infant/Toddler Sensory Profile (ITSP), Infant-Toddler Social and Emotional Assessment (ITSEA), Autism Diagnostic Interview-Revised (ADI-R), and Autism Diagnostic Observation Schedule-Generic (ADOS-G), revealed that toddlers with ASD show higher under responsiveness (described as low registration by the authors) and stimulus avoidance as well as low frequency of seeking behaviors compared to IQ-, age-matched controls (79). Foss-Feig and coworkers investigated both under- and over-responsiveness to tactile stimuli in children with ASD through three measures of sensory processing: Tactile Defensiveness and Discrimination Test-Revised (TDDT-R), the Sensory Experiences Questionnaire (SEQ), and the Sensory Profile (SP). They reported that heightened levels of tactile seeking behavior were associated with more severe levels of social and repetitive behaviors. Additionally, heightened levels of hypo-responsiveness to tactile stimuli were associated with more severe levels of social and non-verbal communication impairments as well as increased repetitive behaviors. Conversely, over-responsiveness was not correlated with any of core symptoms of ASD (80). Data extracted from experimenter-reports of over-responsiveness, parent-reports of tactile symptoms, and self-reports of pleasantness of texture, showed that children with ASD have superior over-responsiveness scores compared to controls. Moreover, they observed a positive correlation between over-responsiveness and parent-report of tactile symptoms and between over-responsiveness and social impairments. Conversely, pleasantness ratings were inversely related with impaired communication (81). However, the contribution of ASD comorbidities such as intellectual disability (ID) and/or language impairments might have a role in defining the responses to studies involving self-reports and have to be considered. An individual with ASD and ID may have difficulties in describing to the experimenter the sensations generated by the stimulus, adding complexity to the interpretation and replication of studies. A risk of an imbalanced picture of ASD may arise and a selection bias for intellectual disability has been reported as issue in ASD research (82, 83). Most of studies on tactile processing so far have focused on children, however there are also studies (4, 84) showing that abnormal sensory processing is also present in adults.

These studies, although informative indicators of the tactile abnormalities in ASD, appear to be inconsistent with respect to pattern of response, correlation among measures, and diagnostic terms. In addition, different types of reports were used in different studies. All such aspects render these studies difficult to be compared; moreover, they do not always correlate to clinical observation, nor they provide indicators of possible cortical dysfunctions.

More recently, researchers have preferred a psychophysics approach to study tactile functionality in ASD in a more objective modality. Some of these studies have shown how detection of tactile stimuli is impaired in both adults and children with ASD (for example in vibration detection; (85), so in line with previous reports. However, other studies showed that tactile detection is normal in autism (57, 86, 87). It is possible to speculate that these differences result from the different type of stimulation used (i.e., flutter, vibration, sinusoidal, or constant) as well as its location. Although these works have the merit of bringing a greater objectivity to the study of tactile abnormalities in ASD, it remains unclear whether underlying sensory mechanisms are altered, or it is the emotional response to sensory input that leads to issues in filtering of the signal resulting in hyper/hypo-responsiveness.

Imaging studies have also tried to investigate the underlying neural mechanism of abnormal tactile sensitivity in ASD. Since tactile stimuli are part of the somatosensory world and as such rely on subcortical and cortical brain regions, researchers focused on possible differences in these brain areas between ASD and TD control subjects. Coskun and colleagues were among the first to report abnormalities in the sensory map organization of ASD individuals. Using magnetoencephalography (MEG) recordings, these authors examined the cortical responses to passive stimulation of the thumb and index finger of dominant hand as well as the lip from ASD and TD controls. They found a different cortical representation of the thumb and the lip in ASD individuals compared to TD controls (88), namely the distance between the cortical representations of these two body parts was significantly larger in the autism group than in TD subjects. Moreover, in the cerebral cortex, the thumb is typically closer to the lip than the index finger; this was not observed in ASD individuals. However, as found in a successive study by the same group (89), the variability of the evoked potential as a response to passive stimulation of the thumb and index finger did not differ between controls and adults with ASD. Conversely, other authors showed a lower amplitude of contralateral cortical S1 response to tactile stimulation in children with ASD (27). Although these studies provide us with useful indications of cortical function in autism, discrepancies exist across studies. Moreover, the variability in neural responses appears to be higher in ASD (90, 91). A possible explanation could be sought in the type of stimulation involved (i.e., passive vs. active) as well as in the high heterogeneity of ASD (2). In addition, a limit of these studies lies in the complexity to compare findings in children with those obtained in adolescents and adults.

Several studies suggest that ASD pathogenesis might involve an imbalance between excitation and inhibition (E/I imbalance). This hypothesis is supported by several lines of evidence showing that the γ-aminobutyric acid (GABA) system is altered in ASD, and that may relate to alterations in sensation and symptoms in both animal models and humans. A pivotal role of GABAergic dysfunction in ASD was first hypothesized in early 2000s by Hussman (92) and Rubenstein and Merzenich (93), even if the key role of GABA in shaping neural response to tactile stimulation (94, 95), as well as in brain development and cortical plasticity (96, 97), was known from many years. Several genetic, neuropathological, and neuroimaging studies showed that GABAergic dysfunctions occur in ASD (98), and defective GABAergic neurotransmission has been suggested as a potential candidate in sensory deficits in ASD (99). In the tactile domain, a study investigating tactile detection thresholds in TD children was the first to report that tactile sensitivity was associated with *GABRB3* genetic variation in typically developing children (100), confirming findings from animal model studies. The *GABRB3* gene, coding for the β3 subunit of the GABA receptor channel, is one of the many candidate genes to be associated with autism (101, 102). Moreover, GABA levels were shown to be reduced in the sensorimotor cortex and positively correlated with worsened detection thresholds in children with ASD; in addition, GABA levels were not correlated with adaptation or frequency discrimination as for TD children (103).

Taken together, these results suggest that altered inhibition could explain some of the behavioral features of tactile abnormalities in ASD. Studies performed in appropriate mouse models contributed to better understand the neurobiological bases of tactile abnormalities in ASD.

## Understanding the Impact of Somatosensory Function in Shaping Social Behavior

Altered sensory processing has revealed to be an important feature for the clinical description of ASD. As discussed above in the review, sensory dysregulation encompasses multiple modalities (vision, hearing, touch, olfaction, gustation) and arises early in the progression of ASD. There is evidence that this could impact social functioning. It has been proposed that sensory stimuli and social behaviors may have a reciprocal influence on each other throughout development (104). This idea is reinforced form findings of early abnormal sensory sensitivity to stimuli predicting later joint attention and language development (18) and higher levels of social impairment in adults with ASD (105).

Touch is considered one of the most basic ways to sense the external world (106) and has been reported to have a significant role in role in several social aspects such as communication (107), developing social bonds (108), and overall physical development and connectivity of brain areas (109, 110). For this reason, skin has been proposed by some authors as “social organ” (111).

It has been suggested that irregularities in touch and tactile perception may be associated with broad levels of social dysfunction in ASD. For example, as described earlier in the paper, touch seeking behaviors have been found to predict levels of social impairment, and tactile hypo responsivity was associated with both poorer social functioning and nonverbal communication skills (80). Differences in tactile processing and tactile preference behaviors in ASD have also been reported in early infancy (112). Furthermore, lack of social touch can lead to higher levels of anxiety, stress, and depression (113), aspects which are commonly seen in ASD population (114, 115). Moreover, atypical touch during infancy can develop into critical deficits later in life, specifically in regards to attachment. While individuals with ASD are capable of forming a secure attachment to their caregivers, they tend to be less securely attached than their typically developing peers (116). In addition, individuals with ASD who have secure attachments tend to have less socially severe symptoms than individuals with ASD who are not securely attached, suggesting symptom severity and overall level of functioning could impact the strength of attachment (117).

Touch is also important in developing social bonding. Oxytocin, the neuropeptide primarily involved in social bonding, has long known to be released in response to positive tactile stimuli (touch, warmth, odors) (118). In individuals with ASD, oxytocin abnormalities have been found in plasma levels (119), in the gene that encodes for the oxytocin receptor, OXTR (120), as well as in oxytocin receptors (121). However, the behavioral and neural effects of oxytocin were negatively correlated with ASD-like traits, suggesting these effects to be diminished in individuals exhibiting low social and emotional abilities associated with autistic traits (122). Future research should look further into the importance of tactile perception in shaping social aspects, as well as its impact on other social domains not previously explored.

Theoretical models have been proposed to integrate sensory and social features of ASD. One model that tried to explain altered sensory functioning in ASD is the “temporal binding hypothesis” (123). This theory lays on the assumption that sensory stimuli that occur in close temporal proximity are more likely to be integrated and so to be perceived as a whole; thus, timing information is crucial to binding and integrating associated stimuli (124). The possibility of an extended “temporal binding window” in individuals with ASD which may give rise to alterations in sensory processing has been proposed (125). Indeed, a longer temporal binding window could create a blurred, unpredictable sensory environment, as unrelated stimuli become bound together. Ideally, throughout development important social cues may fail to become integrated or salient. Thus, according to this theory an extended temporal binding window could negatively impact social behavior in ASD through altered binding of social cues.

Another theory is the “intense world theory” which offers a neurological mechanism for how the sensory and social features of ASD may be related (126, 127). This theory proposes an excessive functioning of neural circuits as the base of sensory and social impairments. Thus, such neural circuits are hyper-reactive, hyper-plastic, and generally up-regulated. This would create an intense world, a fragmented world (with focus on individual components of the environment), and an aversive world. Low level sensory perception is enhanced (intense world) and coupled with deficits in sensory integration (fragmented world). Throughout development, this could lead to an over-specialization for perceiving primary sensory cues at the expense of the ability to navigate in a socially complex world (127). In this way, the intense world theory explains both the unique sensory and the social features of ASD and offers a mechanism for how an up-regulation in primary sensory perception results in social avoidance and withdrawal.

Another theory focus on “atypical hierarchical information processing” as base of sensory and social functioning defects in individuals with ASD. Since we live in a world buzzed with stimuli, in order to adequately perceive and operate in it, humans use both incoming sensory information (bottom-up processes) and inference from prior experience and context (top-down processes) (128). It has been suggested that under-utilization of top-down processes such as context or experience (129) or an over-reliance on bottom-up sensory perception (130) is characteristic of perception in ASD. At the neural level, this profile may reflect hyper-activation of primary sensory cortices, decreased prefrontal activity, and reduced neural habituation during sensory processing (131). According to this theory, this information processing profile may inhibit social functioning as the interpersonal world demands strong central coherences, integration of context, and utilization of prior knowledge. Thus, over-functioning of bottom-up sensory processing coupled with under-utilizing top-down perception in ASD could explain both enhanced sensory processing and inefficient social functioning in this population.

When discussing dysfunctions of the somatosensory system, it is important to consider the sensory processing cascade in its entirety. Starting from the periphery (i.e., the skin, where the mechanical stimuli are transduced in electrical signals), moving to the intermediate stations (i.e., spinal cord and/or brainstem, where the electrical signals are delivered by means of neuronal ascending pathways), reaching subcortical and cortical brain areas (i.e., primary somatosensory cortex and other higher function somatosensory processing areas, where integration/codification of the information occurs), sensory information can undergo more or less severe modifications. Indeed, abnormal development or interaction in any of these steps could ideally lead to abnormal sensory processing. Moreover, since proper tactile perception is of importance in early development as well as in forming social and physical relationships (132), a possible relation between tactile abnormalities and social behaviors could be a matter of fact. For this reason, when assessing the behavioral outcomes of relevant social/sensory task performed by mouse models of ASD, it is at least necessary, when possible, to correlate the behavioral response to a potential neurobiological defect. Indeed, even though humans and animals have evolved under different evolutionary pressures making social behaviors much harder to compare, molecular and cellular functions are strongly conserved and so appear to be mostly comparable.

However, what must be kept in mind is that social behaviors not a unitary behavior with a unique neurobiological basis, but rather different aspects of social behavior show different neural substrates. Moreover, the modulation of environmental cues, the type of sensory stimulation, and the role of conspecific actions in shaping the social response add complexity to our understanding of social behavior in animals (included humans) (133).

In recent years, social neuroscience has made great progress in identifying the neural substrates of social behaviors, and the brain processes linked to social interactions in disease have received considerable attention. In humans, social cognition differentiates between social perceptual processes (devoted to the detection and the analysis of social stimuli like a face), social attribution processes (involved in the inference of other’s and one’s mental states from behavior), and social categorization processes (involved in the process by which individuals are placed into groups based on common characteristics like gender and ethnicity). On the neural level, the social perceptual processes include the primary sensory areas as well as more specialized regions like the fusiform face area (FFA) and the temporo-occipital associative cortex (V5 and extra-striate body area, EBA). The social attribution processes include the premotor cortex, the superior temporal sulcus (STS) and the temporo-parietal junction (TPJ). The social categorization processes encompass the medial/dorsolateral prefrontal cortex and the anterior cingulate cortex. Instead, the emotional content and the motivational appraisal of social stimuli appear to be mediated by the amygdala, the orbitofrontal cortex, and the hippocampus (134, 135). Although one should be always cautious in comparing social behaviors in humans and in mice, it interesting how brain regions such as the amygdala and the hippocampus have been also related to social circuits for behavioral decision in mice (133).

How does social behavior relate to sensory stimulation processing? This question is far from being fully answered, however what we can say is that the sensory inputs (coming from other individuals or from the environment), whether they are olfactory, visual, or somatosensory, are processed and integrated over time with social internal states and transformed into behavioral outputs that in turn provide sensory cues to the other individual forming a feedback loop (133). As an example, somatosensory stimulation is critical for both mating (during copulation) and parenting (tactile stimulation of pups affects the development of normal behaviors) (136, 137).

The hypothesis that peripheral nervous system dysfunctions (namely, dysfunctions of the sensory system) could contribute to ASD pathophysiology has a recent history. Although somatosensory abnormalities in humans and rodents have long been reported, still little is known about their role in ASD. Further efforts are necessary to unravel the neural correlates of social behaviors, and their relationship with sensory processing abnormalities could be of help in describing the social impairments found in ASD.

## Studying the Neurobiological Basis of ASD Through Mouse Models

It has long been known that ASD has a high degree of heritability: studies on monozygotic twins revealed a peak of concordance of 90% compared to 10% of dizygotic twins and siblings (138, 139). However, only recent efforts and technological advancements in genetics made it possible to identify a plethora of gene variants associated with ASD. These variants have been found in several hundreds of different genes and cover the entire spectrum of mutations, from single-nucleotide variants (SNVs) to copy number variants (CNVs), including inherited as well as *de novo* mutations (140, 141). Several genetic mutations in ASD have been associated with genes coding for proteins involved in synaptic functions, such as *SHANK* (142), *CNTNAP* (143, 144), *NLGN* (145, 146), and *NRXN* (147). Some examples of CNVs associated with ASD include chromosomal loci 15q11-q13 (148), 16p11.2 (149), and the *UBE3A* (150), *NRXN1* (147), and *CNTN4* (151) genes. In adding complexity to the understanding of ASD pathophysiology, a subset of single gene mutations associated with ASD are also responsible for other neurodevelopmental disorders, including *FMR1* in fragile X syndrome, *TSC1* in tuberous sclerosis, and *MECP2* in Rett syndrome. The tremendous progress made in identifying all these genes associated with ASD has subsequently resulted in the generation of several ASD mouse models, through which it is possible to infer the effect of single mutations, thus advancing our understanding of the biological bases underpinning this complex syndrome. A multitude of mouse models have been generated by knockout and knockin mutations in ASD candidate genes. In developing new mouse models it is important to consider different aspects such as face validity (i.e., resemblance to human symptoms), construct validity (i.e., similarity to the causes of the disease), and predictive validity (i.e., expected responses to treatments that are effective in the human disease), with the best animal model keeping together the three validity criteria (152).

Given the complex phenotypic and genetic heterogeneity of ASD, developing a mouse model keeping together all these aspects represents a challenge for every researcher. Nonetheless, according to the Simons Foundation Autism Research Initiative (SFARI) Gene database (http://gene.sfari.org/, as of October 29, 2019) there are up to now 264 genetic, 42 pharmacologically induced and 4 inbred mouse models of ASD.

Since the diagnosis of ASD is mainly given by the analysis of behavioral aspects rather than physiological criteria, and being the mice, like humans, a social species displaying an extensive variety of social behaviors, neuroscientists tried to develop and refine behavioral paradigms that could be relevant to the human condition. The symptoms however may be uniquely humans and are often highly variable among individuals, so it appears clear that designing mouse behavioral assays relevant to autistic symptoms represents a unique challenge. However, different behavioral paradigms have been developed considering the two core symptoms of human disorder (social/communication defects and repetitive behaviors) and revealed to be qualitatively efficient and reproducible. For a detailed discussion of these experimental approaches, the reader is referred to the comprehensive review by Silverman et al. (153).

Beyond the central vs peripheral dysfunction dichotomy in ASD, it is interesting how sensory impairments in ASD do not only correlate to tactile processing defects but rather represent a complex multifaceted sensory phenomenon that encompass also other sensory systems. A brilliant example comes from the recent work by Goel and colleagues (154) focused on the sensory processing of *Fmr1^-/-^* mice in the visual domain. These mice exhibit a delayed learning in a visual discrimination task, an impairment similar to the human deficit in visual perception in FXS individuals. The reduced number of orientation-selective pyramidal cells of the primary visual cortex (V1) might represent the neural correlate of this defect. In targeting the visual cortex, the authors also found a reduction in the functional output of parvalbumin (PV) neurons (a subclass of GABAergic interneurons) in *Fmr1^-/-^* mice, as compared to wild-type controls. Surprisingly, when a DREADD (Designer Receptors Exclusively Activated by Designer Drugs) strategy was used to restore PV activity and orientation tuning in V1, *Fmr1^-/-^* mice accelerated learning in the visual task. Other studies focusing on the visual domain has also been carried out on *En2^-/-^* (155), BTBR (156), and *SERT-Ala56* knockin mice (157).

## Organization of the Mouse and Human Somatosensory System

**Figure 1:**
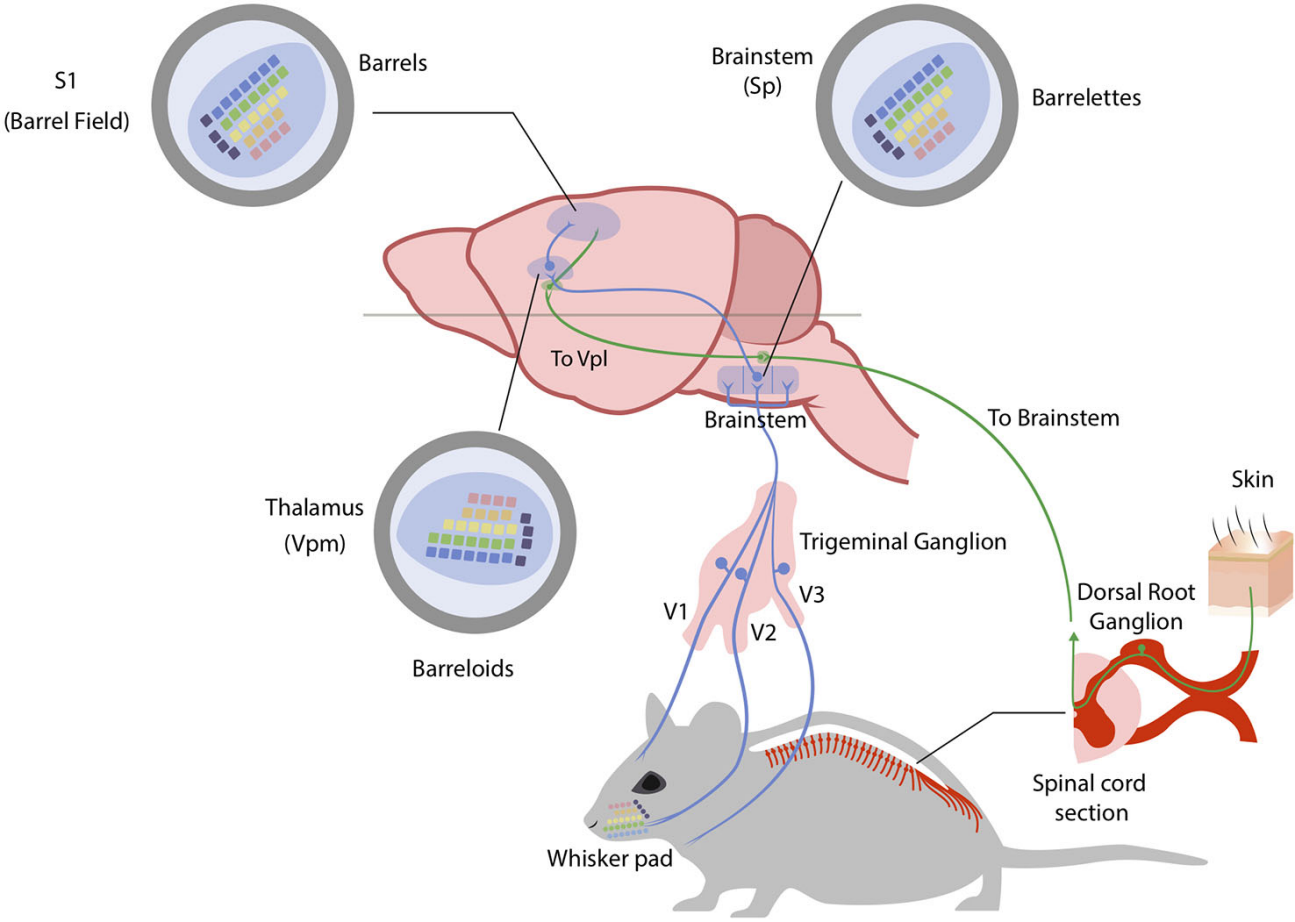
The mouse somatosensory system. Somatosensory stimuli coming from the head region of the mouse are conveyed to the brain through trigeminal ganglion neurons. Neuronal fibers are depicted in blue (for trigeminal ganglion pathway) and green (for anterior and lateral spinothalamic pathways). The ophthalmic (V1), maxillary (V2) and mandibular (V3) branches of trigeminal ganglion process region-specialized somatosensory information with the maxillary branch (V2) innervating the whiskers. Here whiskers are indicated and color-coded to best follow their brain representations (whisker pad). Trigeminal ganglion neurons project to brainstem nuclei (spinal trigeminal nuclei – Sp) where they form an inverted neuronal representation of single whiskers (barrelettes). Trigeminothalamic fibers in turn project to the ventral posteromedial nucleus in the thalamus (Vpm) where again single whiskers are represented and shifted in orientation (barreloids). Finally, thalamocortical axons from the Vpm reach the primary somatosensory cortex (S1) in the barrel field, forming the final neuronal representation of single whiskers (barrels). Somatosensory stimuli coming from the body of the mouse are instead conveyed to the brainstem through dorsal root ganglia (DRG) neurons. The main difference in this system is the fact that somatosensory stimuli are conveyed to the ventral posterolateral nucleus of the thalamus (Vpl) before reaching the sensory cortex. See text for references.

The somatosensory system in mammals conveys sensory information from receptors located in the skin, muscle, and joints to the brain. In mice, the somatosensory system is dominated by the input coming from the facial vibrissae: the neuronal representation of whiskers in the primary somatosensory cortex (the barrel field) occupies more than two thirds of its total area (158). The anatomical and functional organization of the somatosensory system is highly conserved and is based on two major ascending components: the dorsal column system and the trigeminal system. The first-order sensory neurons are the dorsal root ganglion cells and the trigeminal ganglion cells that collect information from the receptors located in the body and the face, respectively. The whisker pad of mice is highly innervated: a single whisker follicle is sheathed in a complex capsular structure which receives up to 200 axonal projections (159). Moreover, the nerves of a single whisker do not connect with the adjacent follicle (160). The dorsal root ganglion (DRG) neurons send their central processes to make synapse in the spinal cord while the trigeminal ganglion cells make synapse in the hindbrain. The main hindbrain nucleus receiving afferents from the whisker system is the spinal trigeminal nucleus (Sp). The Sp can be divided in the oral, interpolar and caudal part (Sp5O, Sp5I, Sp5C), forming the largest nucleus of the mouse hindbrain. The whisker macro representation starts to be appreciable at the level of the hindbrain in concrete structures called “barrelettes” (161). The spinal cord and hindbrain nuclei in turn project to specialized somatosensory nuclei of the thalamus: the ventral posterior group (VP). The initial anatomical separation of the two systems is interrupted at the level of the thalamus, which represents a relay station for all sensory stimuli. The VP region of the thalamus is subdivided into a large medial portion (VPM), which receives afferents from the trigeminal system, and a smaller lateral portion (VPL) which instead receives afferents from the limbs and the trunk. The size of each subdivision of VP is proportional to the number of afferents, so the VPM appears to be larger than the VPL. Moreover, even from the VPM it is possible to appreciate a representation of individual facial whiskers, the so called “barreloids” (162). Somatosensory processes also terminate in clusters of heterogeneous thalamic nuclei (the posterior group, Po) lying medial, dorsal, and caudal to VPM. The largest component of the Po forms the medial subdivision (PoM), which also receives inputs from the whisker pad providing a parallel source of information to the primary somatosensory (S1) cortex (163). In rodents, two further clusters of nuclei have been identified in this region of the thalamus: the reticular nucleus of the thalamus (Rt) and the zona incerta (ZI). These two clusters do not receive somatosensory input from the brainstem or spinal cord but being packed with GABAergic neurons and strongly projecting to the VP, they are thought to play an important role in modulating the output of VP (164). All somatosensory stimuli converge onto the primary (S1) and secondary (S2) somatosensory cortices. S1 is dorsolateral in the rostral part of the neocortex, whereas S2 is located laterally to S1. The primary somatosensory cortex in mice is dominated by the barrel field (S1BF), containing the representation of single facial whiskers. In 1970, Woolsey and Van Der Loos were the first to report these distinct anatomical structures named “barrels” (165). Further division of the S1 are the forelimb area (S1FL), the trunk area (S1Tr), and the hindlimb area (S1HL), with each of these areas characterized by a thick condensed layer IV. **Figure 1** schematically reports the organization of the somatosensory pathways in mice.

**Figure 2:**
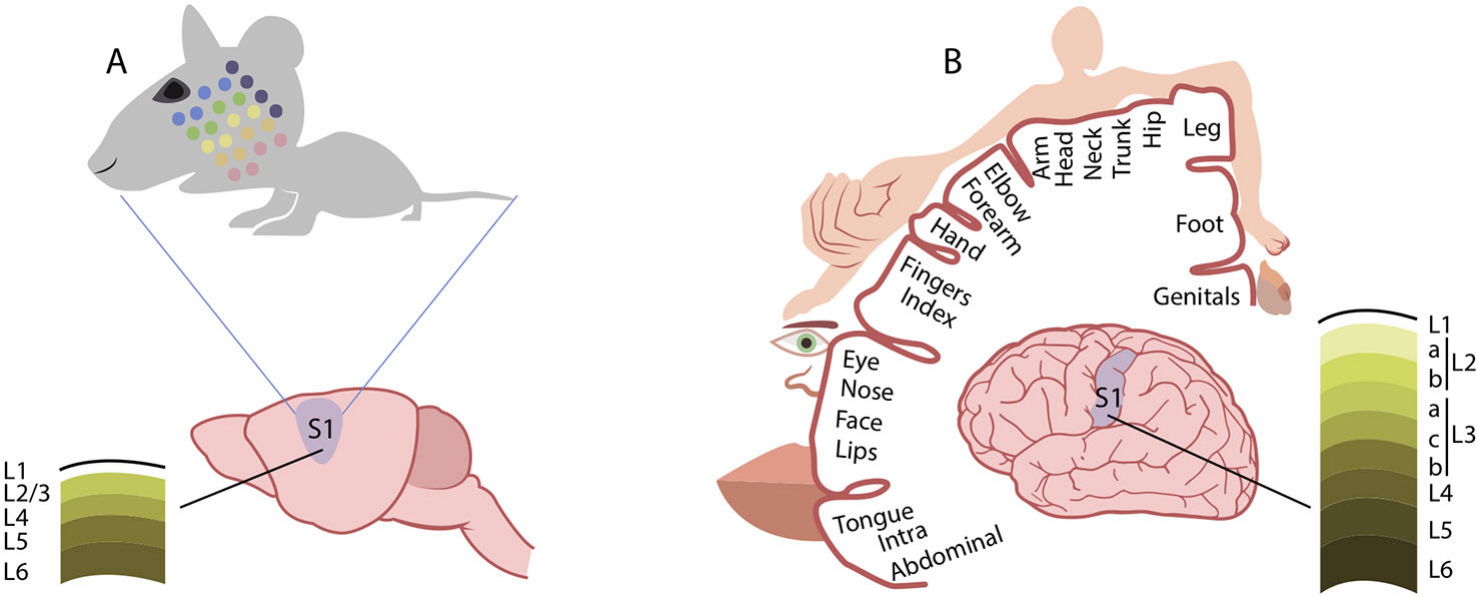
Comparison of cortical somatosensory representation in mice and humans. Distorted representation of body areas in the mouse **(A)** and human **(B)** primary somatosensory cortex (S1). In both species, S1 somatosensory maps reflect the extent of cortical areas devoted to the processing of sensory information from different parts of the body. In mice, the altered proportions of the head and whisker pad with respect to other body regions mirrors the extent of innervation from these areas. Similarly, in humans, the cortical somatosensory representation is enlarged for those regions, such as the hands and the lips, that are densely innervated by sensory fibers. Conversely, the structure of supragranular layers 2 and 3 markedly differs between the mouse **(A)** and human **(B)** somatosensory cortex. See text for references.

As compared to the mouse, the human somatosensory system presents important similarities and differences. Somatosensory receptors located in the skin are essentially the same, and the anatomy of the ascending pathway organization is maintained in both species. The organization of somatosensory cortex found in mice is comparable to that found in mammals with relatively little expansion of the neocortex (166). Much of somatosensory cortex in these mammals is represented by two distinct systematic representations of the contralateral body surface, named the first (primary) representation, or S-I, and the second representation, or S-II (167). The larger S-I represents the body from tail to mouth in a mediolateral cortical sequence, while the smaller S-II has a head-to-tail mediolateral (or dorsoventral) cortical sequence (168). Instead, somatosensory cortex in higher primates (including humans) contains more subdivisions than somatosensory cortex in non-primates. Experiments on the organization of anterior parietal cortex in macaque monkeys defined S-I as a broad region including cytoarchitectonic areas 3 (3a and 3b), 1, and 2 of Brodmann, though Kaas argues that only area 3b should be considered primary somatosensory cortex (168, 169). Area 3b, indeed, forms a complete representation of the body surface. In mice, two whiskers that are adjacent to each other on the animal’s face are represented in adjacent cortical barrels, and the barrel field constitutes a topographic map. Similarly, a topographical organization of the somatosensory cortex (the so called homunculus) is present in humans (170). As for the cortical representation of the whiskers in mouse and rat, the homunculus is a topographic map because neighboring sites on the skin are represented at neighboring sites in the cortex. The whiskers are the critical touch organ in rats and mice, whereas in humans and other primates the fingertips are their equivalent. Each fingertip is innervated by axons from 250–300 sensory neurons (a comparable number as the whisker) and because individual axons terminate in multiple receptor structures, the density of mechanoreceptors is remarkably high (over 1,000 per cm^2^). One important way in which fingerprint touch differs from whisker touch is that primates manipulate objects with our hands whereas rodents do not manipulate objects with their whiskers. This difference is evident when comparing the mechanism for sensing texture. For mice and rodents in general, the firing rate of neurons in barrel cortex differ from rough to smooth surface (171). In primates, the perception of coarse textures is based on the difference in firing rate between adjacent slowly adapting neurons(172); the perception of fine surfaces is based on vibrations in the skin, transduced by rapidly adapting Pacinian receptors (173). Finally, important differences have been found in the structure of supragranular layers 2 and 3 of the mouse and human somatosensory cortex (174). **Figure 2** schematically reports the somatotopic representation of the mouse and human primary somatosensory cortex.

## Somatosensory Abnormalities in Mouse Models of Autism-Risk Genes

The scope of this review is to focus on sensory abnormalities in genetic mouse models of ASD (that is, mice bearing mutations in ASD-relevant genes). Alternative, non-genetic models such as maternal immune activation and valproate exposure during pregnancy have revealed to be valuable tools to study ASD-like phenotypes in rodents (175, 176). These studies contributed to the neurobiological investigation of sensory abnormalities in ASD (177, 178), but will not be reported in detail in this review.

ASDs are generally thought to be caused by defective brain development, and most studies traditionally focused solely on brain alterations. However, emerging evidences from mouse studies suggest that at least some aspects of the disorder are linked to defects in the peripheral nervous system that communicates sensory information to the brain. **Table 1** summarizes the most relevant mouse models of autism-risk genes, described in this section, which display somatosensory system defects at central or peripheral level. For an extensive summary ASD-relevant mouse strains showing somatosensory deficits, the reader is referred to the AutDB database (http://autism.mindspec.org/autdb). A brilliant example of central sensory processing defects comes from the work of Michaelson and colleagues (179). Starting from the finding of touch-related sensory processing defects (i.e., blunted responses to painful touch-related stimuli and/or tactile seeking behavior as well as tactile aversive behaviors) in *SYNGAP1* heterozygosity in humans, they found that *Syngap1* heterozygosity causes touch-related deficits in cortical circuit activation in mice, namely a reduced whisker related activation of receptive fields in the primary somatosensory cortex. Moreover, alteration of whisker evoked activation was found to be whisker-dependent. Particularly, neurons in the layer 2/3 of the somatosensory cortex were less active in heterozygous mice compared to WT in live calcium imaging experiments. Interestingly, these deficits in touch-related cortical circuits were associated with reduced whisker-evoked synaptic potentials in layer 2/3 and anatomical irregularity of layer 4 neurons. This is interesting because neurons of layer 2/3 of the somatosensory cortex integrate bottom-up sensory signals originating in the periphery with information arriving from higher cortical areas (186), whereas neurons in layer 4 receive the bulk of sensory-related information arriving from subcortical areas (187). So, ideally these defects could represent the neurological basis of tactile behavior abnormalities. Indeed, the authors found that *Syngap1* heterozygous mice were unable to discriminate among objects that differ for the texture in the NORT test. Thus, a possible correlation among circuitry dysfunctions and tactile behavior deficits could be a matter of fact.

**Table 1:** Somatosensory deficits in mouse models for autism-risk genes.

Mouse model	Behavioral test	Measure	Phenotype	Age at testing	Sex	Reference
*Syngap1^+/-^*	Tactile novel object recognition test (NORT)	Time spent exploring the novel object	Increased in HET	6-8 weeks	M/F	179
	Go/no go task involving whisker deflection	Correct answers reporting whisker deflection	Decreased in HET			
*Fmr1^-/-^*	Tactile defensiveness assay in head-restrained mice	Withdrawal/habituation to whisker stimulation	Increased in KO	PD 14-16 and 35-41	M/F	180
*Shank2^-/-^*	Electronic von Frey Apparatus	Mechanical withdrawal threshold	Increased in KO	>2 months	n.s.	181
	Hot plate test	Latency to first reaction	Increased in KO			
	Von Frey filaments for allodynia after neuropathic or inflammatory pain	Positive response (licking, biting, withdrawal)	Decreased in KO			
*Cntnap2^-/-^*	Calibrated von Frey hairs	Mechanical withdrawal threshold	Decreased in KO	8-16 weeks	M/F	182
	Hot plate test	Latency to withdrawal	Decreased in KO (53°C)			
	Pain sensitivity to capsaicin and formalin	Duration of response (licking, biting, paw lifting)	Increased in KO			
*Mecp2* KO, *Skank3B* HET, and *Fmr1* KO	Texture specific novel object recognition	Preference for the novel object	Decreased in all models	6-8 weeks	M/F	183
*Mecp2* KO, *Skank3B* HET, and *Fmr1* KO	Tactile prepulse inhibition assay (T-PPI)	Air puff response	Increased in all models	6-8 weeks	M/F	183
*Mecp2 cKO in DRG*	Texture specific novel object recognition	Preference for the novel object	Decreased in cKO	6-8 weeks	M/F	183
	T-PPI	Air puff response	Increased in cKO			
*Gabrb3* HET and *Gabrb3* cKO in DRG	Texture specific novel object recognition	Preference for the novel object	Decreased in both models	6-8 weeks	M/F	183
	T-PPI	Air puff response	Decreased in both models			
*Shank3B* HET and *Shank3B* cKO in DRG	T-PPI	Air puff response	Increased in both models	6-8 weeks	M/F	184
	Texture specific novel object recognition	Preference for the novel object	Decreased in Shank3B HET			
*En2^-/-^*	Whisker nuisance test	Scoring of avoidance behaviors	Increased in KO	3-6 months	M/F	185

cKO, conditional knockout; DRG, dorsal root ganglia; F, female; HET, heterozygous; KO, knockout; M, male; NORT, tactile novel object recognition test; n.s., not specified; PD, postnatal day; T-PPI, tactile prepulse inhibition assay.

This possibility is supported by the research carried out by He and colleagues on the *Fmr1* knockout mouse model of ASD (180). Fragile X syndrome (FXS), in which transcriptional silencing of the *Fmr1* gene leads to loss of the fragile X mental retardation protein (FMRP), represents one of the most common single-gene cause of autism [from 1% to 6% of cases; (188)], and the vast majority of FXS individuals show tactile impairments (189). The *Fmr1* knockout mouse model of FXS exhibits behavioral deficits analogous to human symptoms and, as reported by He and colleagues, also shows tactile defensiveness measured as avoidance motor response in a whisker stimulation test in both juvenile (P14-P16) and adult (P35-41) mice. Moreover, the authors reported that in young mice only a reduced fraction of neurons of layer 2/3 of the barrel field were responsive to whisker stimulation in a time-locked manner and showed impaired adaptation to repeated whisker stimulation, suggesting that this could represent the explanation for the observed behavioral over-reactivity (180).

Another study focused on behavioral aspects of sensory processing, showing reduced nociception and chronic pain in *Shank2^-/-^* mice (181), as an extension of tactile hyposensitivity found in ASD individuals. These authors reported basal tactile sensitivity impairment in *Shank2^-/-^* mice as compared to WT, namely a higher basal mechanical threshold (the force applied when the mouse withdraws its paw) by using an electronic Von Frey apparatus (used to assess withdrawal responses in rodents). Moreover, the authors found a reduced sensitivity in *Shank2^-/-^* mice to chronic neuropathic pain (i.e., induced by nerve ligation) as well as inflammatory pain (i.e. induced by antigens injection) suggesting that these alterations could be due to defect both at the brain level than at peripheral level. Indeed, peripheral synaptic dysfunctions in the spinal cord, as well as central somatosensory cortex defects could explain these impaired responses in *Shank2^-/-^* mice.

Recent studies indicate that peripheral alterations of tactile sensitivity in mouse models of autism-risk genes might contribute to social and sensory behavior defects relevant for ASD. One example comes from the work of Dawes and colleagues on the *Cntnap2* ^-/-^ mouse (182). They found that loss of *Cntnap2* resulted in pain related hypersensitivity (as tested through the Von Frey apparatus and the pinprick application) in mice. Since *Cntnap2* was found to be expressed in dorsal root ganglion neurons (DRG), the authors measured primary sensory neuron activity *in vivo* through calcium imaging and *in vitro* through patch-clamp technique to assess if *Cntnap2* could impact neuronal excitability. They showed that DRG neurons were significantly hyper-responsive to sensory stimulation showing larger increase in intracellular calcium concentration and significantly lower rheobases (defined as the smallest injected current that elicit an action potential) compared to WT. Moreover, they found from *in vivo* extracellular recording of DRG neurons that loss of *Cntnap2* leads to dorsal horn neuron hyper excitability, in line with the behavioral assays.

In line with these findings, Orefice and colleagues showed that mice harboring mutations in *Mecp2*, *Gabrb3*, *Shank3*, and *Fmr1* genes exhibit aberrant tactile sensitivity, as detected by abnormal behavioral responses to skin or whisker stimulation (183). When compared with control wild-type littermates, all these mutant mice failed to distinguish between smooth and rough-textured objects in the texture novel object recognition test (NORT), indicating impairments in skin-based texture discrimination. In addition, this study tested sensorimotor gating and skin sensitivity using the tactile prepulse inhibition test (PPI), which consists in delivering puffed air onto the back of mice and evaluating whether this prepulse could inhibit a subsequent startle response to a loud stimulus. Interestingly, all mutant mice tested showed an enhanced response compared to controls. Further testing additionally demonstrated that this exaggerated response was elicited by air puff alone, suggesting an abnormal hypersensitivity to tactile stimulation (183). In order to explore the neuronal basis of this tactile deficit in *Mecp2* mutant mice, the authors deleted *Mecp2* in different body areas, namely from forebrain excitatory neurons, from the neurons caudal to cervical level 2 (including the spinal cord and the peripheral sensory system), and from the sensory ganglia (including trigeminal ganglia). Sensory testing through NORT and air PPI revealed that somatosensory deletion of *Mecp2* alone leads to aberrant tactile sensitivity. The authors then tested the hypothesis that GABA imbalance could have a role in impaired tactile sensitivity in *Mecp2* and *Gabrb3* mutant mice: deletion of *Mecp2* or *Gabrb3* in peripheral somatosensory (dorsal root ganglia, DRG) neurons caused mechanosensory dysfunction through loss of GABA_A_ receptor-mediated presynaptic inhibition of inputs to the CNS (183). More recently, using a similar approach, the same authors found that acute treatment with GABA_A_ receptor agonist selectively acting on mechanosensory neurons reduced tactile over-reactivity in six different ASD mouse models, both genetic and environmental (184). Moreover, chronic treatment of two genetic mouse lines, namely *Mecp2* and *Shank3* mutants, improved multiple ASD-associated behavioral phenotypes such as tactile over-reactivity, anxiety-like behaviors and social impairments. These results strongly support the hypothesis that peripheral somatosensory circuit dysfunctions could contribute to social deficits in ASD.

The idea of GABA imbalance in explaining the somatosensory defects reported in mouse models of ASD (see above, *Studying the Neurobiological Basis of ASD Through Mouse Models*) comes from the studies on the *Gabrb3* gene, which encodes one subunit of GABA receptors on postsynaptic neurons and is associated with ASD (102). Mice heterozygous for this gene show a reduced startle response. In addition, an increased tactile sensitivity and a reduced sensorimotor processing were reported for *Gabrb3* heterozygous male mice (190), and a reduced expression of *Gabrb3* was found in *Mecp2* deficient mice (191). A similar approach was used by our laboratory in describing the somatosensory defects of *En2* mutant mice (185). Genetic studies (192–194) and expression analyses on post-mortem brain tissues (195–197) indicated that deregulated expression of the human *EN2* gene is linked to ASD. Accordingly, *En2^-/-^* mice are considered a reliable model for investigating the neurodevelopmental basis of ASD. Indeed, *En2^-/-^* mice show ASD-like behaviors (198), and a lower expression of *Fmr1* (199) accompanied by anatomical defects common to *Fmr1* knockout mice (200). We reported that *En2^-/-^* mice have a significantly reduced synchronization in somatosensory-auditory/associative cortices and dorsal thalamus, suggesting the presence of aberrant somatosensory processing in these mutants. Indeed, when tested in the whisker nuisance test (201, 202) *En2^-/-^* mice showed hyper-responsiveness to repetitive whisker stimulation. In line with our findings of primary somatosensory cortex functional hypo connectivity, sensory hyper-responsivity in *En2^-/-^* mice was accompanied by a reduced activation of primary somatosensory cortex showed by a decreased c-Fos immunoreactivity in layer IV. Interestingly, whisker stimulation under anesthesia also resulted in reduced *c-fos* mRNA expression in the *En2^-/-^* mice primary somatosensory cortex, corroborating the data obtained following whisker stimulation in freely moving animals. Our hypothesis is that this disruption of sensory processing in *En2^-/-^* mice is likely due to impaired function of GABAergic signaling, since *En2^-/-^* mice present a reduced number of GABAergic interneurons in the hippocampus and somatosensory cortex (203). In addition, altered electrophysiological and behavioral markers of sensory processing can be rescued by pharmacologically enhancing GABAergic signaling in ASD mouse models (98). Further efforts are needed to reveal the anatomical networks by which GABAergic deficits impact somatosensory processing in mice models of ASD. Our current work focuses on exploring the potential somatosensory defects in different sensory areas such as the trigeminal ganglion, the thalamus and the somatosensory cortex trying to extend these findings to other mouse models of ASD.

Together, these findings reinforce the need of studying sensory features of ASD in mouse models and suggest that tactile impairment in mice, akin to human ASD tactile abnormalities, could be explained through sensory processing defects in the peripheral and central nervous system.

## Conclusions

In this review, we have discussed evidences of sensory impairments found in ASD. Abnormal sensory processing in autism represents a common feature and is recognized as a diagnostic criterion. It encompasses many aspects of all sensory systems, leading to both central and peripheral defects. Mouse models of autism-risk genes recapitulate sensory impairments found in autistic individuals and represent a valuable tool to study the cellular and molecular mechanism underlying sensory behaviors. We have addressed the organization of mouse somatosensory system to introduce the most recent findings on tactile sensitivity in genetic mouse models of ASD as well as studies on aberrant sensory processing in somatosensory and other sensory domains. Further efforts are needed to effectively link the sensory abnormalities and social features of ASD to the intrinsic multifaceted nature of sensory dysfunctions in ASD.

## Ethics Statement

Experiments performed in our laboratory and reported in this review were approved by the University of Trento Animal Welfare Committee and Italian Ministry of Health.

## Author Contributions

All authors contributed to draft the manuscript. LB wrote the manuscript. GP and YB edited the manuscript. GP and YB provided funding.

## Funding

GP and YB are currently supported by the University of Trento 2018–2020 Strategic Project “Trentino Autism Initiative – TRAIN”.

## Conflict of Interest

The authors declare that the research was conducted in the absence of any commercial or financial relationships that could be construed as a potential conflict of interest.
